# The Validity of Adding ECG to the Preparticipation Screening of Athletes An Evidence Based Literature Review

**Published:** 2014-12-19

**Authors:** A Alattar, N Maffulli

**Affiliations:** 1 Physical Medicine & Rehabilitation Department, Rashid Hospital, Dubai Health Authority, UAE; 2 Department of Musculoskeletal Disorders, University of Salerno, Salerno, Italy; 3 Queen Mary University of London, Centre of Sports and Exercise Medicine, London, UK

**Keywords:** *Sudden cardiac death*, *electrocardiography*, *physical examination*, *athlete’s heart Syndrome*, *cardiomyopathies*

## Abstract

**Objective:**

To review the available evidence establishing the validity of adding electrocardiogram to the preparticipation cardiac screening in athletes.

**Data Sources:**

MEDLINE and CINAHL databases were searched. Additional references from the bibliographies of retrieved articles were also reviewed and experts in the area were contacted.

**Selection Criteria:**

Only original research articles seeking to establish the use of electrocardiography followed by second line investigations in athletes under 36 years of age were reviewed.

**Search Result and Quality Assessment:**

The initial literature search identified 226 papers. Of these, 16 original articles (all type II evidence—population-based clinical studies) met the selection criteria and directly related to the use of electrocardiography in athletes cardiac screening. The methodological qualities of included studies were assessed using the Downs and Black checklist.

**Conclusion:**

Screening with electrocardiography represents best clinical practice to prevent or reduce the risk of sudden cardiac death in athletes. It significantly improves the sensitivity of history and physical examination alone; it has reasonable specificity and excellent negative predictive value; and it is cost-effective. Future studies must be large, multicentre, multination, prospective trials powered to determine how different screening options affect the incidence of sudden cardiac death. Efforts should also be targeted toward secondary prevention of sudden cardiac death with pitch side cardiac resuscitation and the immediate use of defibrillator.

## 1 Introduction

Sudden cardiac death (SCD) is the leading cause of mortality in young athletes during exercise, and it mainly results from undiagnosed structural or electrical cardiovascular disease [1, 2]. The incidence of SCD in young athletes varies widely from 0.5–2/100,000/year [3,4]. The best way to screen athletes for conditions predisposing them to sudden cardiac death is a topic of debate. In 2005 the European Society of Cardiology proposed a common European protocol for cardiovascular preparticipation screening of athletes for the prevention of the sudden cardiac death [5]; The success of the European model of preparticipation evaluation is mostly attributed to the inclusion of electrocardiography to basic screening protocol and its great ability to identify athletes at risk of sudden cardiac death due to some underlying cardiac abnormalities [6]. On the contrary, the American Heart Association does not endorse the routine use of electrocardiography for mass cardiac screening of athletes on the presumption of high false positive results [7] especially when the screening is merely used as a prevention of sudden death caused by rare and hereditary conditions [8].

This review offers an up to date perspective on the effectiveness of electrocardiography cardiac screening in athletes. The latest review was done in 2004 [9].

## 2 Methods

### 2.1 Inclusion and exclusion criteria

Inclusion criteria: (1) original published studies in English language in peer-review journals involving the use of electrocardiography in preparticipation evaluation of athletes; (2) must include second line investigation (echocardiography, stress ECG, 24 hours ECG etc.); (3) study population not restricted to gender, ethnicity or athletic level, yet age restricted to less than 36 years of age; and (4) no restriction on study design. Studies were excluded if they involved non athletic population.

### 2.2 Search strategy

An electronic database search was performed using MEDLINE (1948 to present) and CINAHL Plus with Full-Text (1937 to present). A keyword search yielded MeSH/CINAHL headings, which were combined and exploded (Table 1). Searches were restricted to English language and human subjects. The list of references was downloaded into EndNote reference manager website, including MeSH headings and abstracts and duplicate references were removed. The bibliographies of the final articles selected were scanned to assure no articles were excluded. The relevant original articles relating specifically to the review question were retrieved. Experts in the area were contacted for relevant and even on-going research.

## 3 Result & Discussion

### 3.1 Review selection and identification

The search strategy retrieved 226 papers, of which 188 were narrative reviews, technical notes and letters/personal opinion. A total of 38 relevant trials underwent further analysis for inclusion. 22 studies were excluded, of which four studies targeted senior athletes [10–13], nine looked at structural and electrophysiological remodelling of the athletes heart [14–22], five studies discussed the aetiology and management of sudden cardiac death [23–27], three studies were surveys examining methods of athletes screening [28–30], and one examined use of echocardiography in cardiac screening [31]. Consequently, 16 original articles met the selection criteria, and directly relating to the use of electrocardiography in athletes cardiac screening (*Table 2*).

### 3.2 Methodological quality assessment

All of these contained type II evidence-population based clinical studies and were considered for methodological quality assessment using modified Downs and Black checklist [32] (Table 3). Recurrent weaknesses were related to issues of confounding, blinding participants and assessors, adjusting analysis for length of follow up, randomization and reporting calculation for sample size.

### 3.3 Studies favouring the addition of electrocardiography

The first mandatory mass cardiac screening in children began in Japan in 1973. The screening process included a questionnaire and an electrocardiography test for all students, regardless of athletic participation. Tanaka et al [33] reported the cardiac screening results of 37,807 young adolescents entering seventh grade. Only three sudden deaths occurred during 6 years follow-up; one 14-year-old boy had hypertrophic cardiomyopathy identified during screening and died while jogging (one death out of 9 high risk identified). The remaining two students, 13 and 16 year old, died during handball and basketball, respectively. Both had a normal electrocardiogram, showing the difficulty in detecting the rare young athletes at increased risk of sudden death during competition even with electrocardiography screening. The author concluded that the cardiac screening system is effective in identifying high risk group and cost-effective (estimated cost of $8,800 per year of life saved). The death of the high risk participant with diagnosed hypertrophic cardiomyopathy might indicate the lack of knowledge of whom to restrict from sports participation. Moreover no autopsy was performed; thus the cause of sudden death cannot be attributed to cardiac causes.

It has been inferred that exercise serves as a trigger for individuals with underlying heart disease and that early detection may lead to prevention via sports exclusion [34]. However, the evidence for this is scarce. One notable exception is the northern Italian experience, were in 1982 routine 12-lead ECG screening in athletes was initiated, and sudden cardiac death rates in the Veneto region of Italy during the next two decades decreased from 3.6/100,000 athlete-years to 0.4/100,000 athlete-year in 2004 [35]. Of the 42386 screened athletes, 8.9% required additional cardiovascular testing. Extrapolating the Italian data to other population is questionable; one can argue that the study was a population based observational report, not a controlled comparison of screening versus non-screening in athletes. In addition, the event rates included all events, not those that occurred only with exertion. In the Veneto region, arrythmogenic right ventricular cardiomyopathy is a major cause of sudden cardiac death, yet in other countries such as USA it is hypertrophic cardiomyopathy. Confounding factors to the 89% reduction in sudden cardiac death might be related to awareness of the community toward the issue and the better use of onsite cardiac resuscitation with defibrillator.

Pelliccia et al [36] tested the diagnostic efficacy of the Italian preparticipation screening program for identifying hypertrophic cardiomyopathy; they performed echocardiography on 4450 athletes already eligible to participate. They found two cases of hypertrophic cadiomyopathy, of which one was diagnosed genetically. Normal electrocardiography had a very high negative predictive value (99.98%) for excluding hypertrophic cardiomyopathy in this 8 years follow-up study. Based on this long term experience and the conclusion that electrocardiographic screening is ‘life saving’ [37,38] the European Society of Cardiology and the Lausanne protocols [5,39] include 12-lead ECG in athlete’s cardiac screening.

Recent publications have provided some insight on the practicalities of electrocardiographic cardiac screening in athletes. Bessem et al [40] included 428 screening test out of 825 screening performed; following a good inclusion/exclusion criteria (age 12–35 and excluding subjects with established cardiovascular conditions) only 6.3% of electrocardiography required further assessment, and electrocardiography had a false-positive screening outcome of 11%. This slightly higher false-positive rate may be partly related to the population screened, with 7% of athletes being referred for screening because of cardiovascular symptoms. When we look at the number of athletes needed to be screened to find a single athlete with a cardiovascular disease, a total of 143 athletes seem acceptable. This study shows that the Lausanne recommendations are an effective screening tool to detect potentially lethal cardiovascular diseases. The Dutch experience showed similar finding to the earlier Italian findings.

Hevia et al [41] showed that the rate of abnormal electrocardiography is low (6.14%) and only 3.27% required additional tests. The value of history and examination alone is questionable, with false positive of 1.2%. This study was a descriptive result of cross sectional study, without providing follow-up data on clinical end point. The sample group was a selected population of male white athletes, and the results should not be extrapolated to other sex or ethnic groups. But the use of well-trained sports physician and cardiologist added strength to this study by minimising the false positive rate of electrocardiography.

Interestingly Sofi et al [42] examined 30,065 subjects and showed that only a small proportion (1.2%) of athletes had distinct abnormalities identified on resting electrocardiography. Thus, false positives were few. Importantly, 153 of the 159 true positives involving athletes disqualified from sport with an identified cardiovascular disorder would have been missed on history and physical examination alone. Thus, adding electrocardiography will detect more athletes with silent cardiovascular disorders at risk of sudden death. Two important issues related to this study; firstly, the cohorts age ranged from 5 to 92 years of age (40% >30 years of age), thus one cannot compare these results to most studies where the cohort were young athletes; secondly there was no second line investigation in participants with positive screening results making it difficult to interpret the real diagnostic power of electrocardiography, as well as the important matter of false positive results.

Wilson et al [43] examined junior athletes and physically active schoolchildren. This study was well conducted, with subjects representing the sporting community and questionnaires needed parent’s completion. The use of a detailed medical questionnaire improved its sensitivity. Moreover, the rate of further investigations was reduced to 4% compared to 8.9% in the Italian experience [37], because electrocardiography was conducted by UK’s leading sport’s cardiologists. The false-positive rate was higher for medical questionnaires than for electrocardiography alone in schoolchildren. The prevalence of junior athletes diagnosed with a cardiac disease was over twice (0.5%) that of schoolchildren (0.2%), this supports the recommendation to screen selected groups to minimise expenditure and improve accuracy of testing. None of the diagnosed athletes were symptomatic, confirming that history and examination alone are inadequate. Hypertrophic cardiomyopathy the commonest cause of sudden cardiac death in young athletes [44] was not detected in this study, although electrocardiography is abnormal in >90% of patients with hypertrophic cardiomyopathy.

Interestingly new supportive data for the effectiveness of adding electrocardiography are coming from United States. Baggish et al [45] compared a screening protocol with or without electrocardiography, and showed that addition of electrocardiography improved sensitivity for detecting serious cardiac abnormalities from 45.5% to 90.9%, and altered the negative predictive value of screening from 98.7% to 99.8%. The false positive rate was 16.9%. These findings were similar to the work of Magalski et al [46], with electrocardiography improving sensitivity of medical questionnaire and physical exam from 44.5% to 88.9% and altering the negative predictive value from 99.3% to 99.9%. The false positive rate were higher in black athletes and might have been reduced overall if the examiners where specialised cardiologists and used the latest European Society of Cardiology electrocardiography criteria [47]. Both studies participants had received preparticipation screening at college already, thus the study cohort may underrepresent the true burden of occult cardiac disease.

Marek et al [48] examined the feasibility of a large-scale high school electrocardiography screening program (Young Hearts for Life [YH4L]) developed in Chicago, USA. This retrospective cohort study examined 32,561 high school students between 2006 and 2009. It involved administrators, cardiologists and community volunteers who underwent specialised training and quality review. Only 2.5% had abnormal electrocardiography requiring further evaluation. These new American findings can have great implications for implementing screening and preventing cardiac sudden death in USA.

These studies clearly showed that history and physical examination alone are inadequate; electrocardiography has an independent added value for diagnosing cardiac disease that can lead to sudden cardiac death. Apart from the Italian experience, most of the studies could not draw definitive conclusions about the effect of the different screening strategies on the incidence of sudden death in athletes.

The latest studies have compared the performance between history, physical examination, and electrocardiography during preparticipation cardiovascular screening (Table 4). The combined average sensitivity from these studies to identify athletes with at risk conditions using history and physical examination is 12% compared with 88% using electrocardiography.

### 3.4 Studies not favouring the addition of electrocardiography

Observational data with the inclusion of electrocardiography in athletic screening in multiple countries have not reproduced the Italian experience. In 1982 Maron et al [49] examined 501 athletes, electrocardiography showed specificity of 27% and high false positive of 15%. The conclusion was drawn that electrocardiography is poorly sensitive. One can argue that the “cardiac outcome” in this study was ill defined; they looked for hypertrophic cardiomyopathy, Marfan syndrome and cardiovascular disease. The cohort was too small to detect rare causes of sudden cardiac death.

Pelliccia et al [50] examined 1005 athletes; he recorded electrocardiography sensitivity of 51%, specificity of 61%, positive predictive value of 7%, and negative predictive value of 96%. The cohort was heterogeneous; the first group underwent routine cardiac screening while the second group where specifically referred to the center for suspected cardiac conditions. Highest incidence of heart disease (15%) was seen in the referred group. Thus this data cannot be compared directly to other large electrocardiography screening studies; moreover one cannot determine what value electrocardiography added beyond history and examination alone.

Fuller et al [51] cohort was larger (5615 high school athletes); addition of electrocardiography improved the sensitivity of screening to 70%, while echocardiography increased it to further 80%. Electrocardiography had similar specificity to history and examination alone, yet more effective screening tool with a false positive rate of 2.7 % (2000 athletes had further tests). The true incidence of cardiac disease is not known in this cohort, because echocardiography was not performed in the entire cohort.

Congenital coronary artery anomalies could be missed in the absence of imaging techniques, Basso et al [52] examined 2 large registries of Sudden Cardiac Death in young athletes in USA and Italy, all of the 27 deaths related to Anomalous Origin of Coronary Artery had normal electrocardiography, echocardiography and stress test. Thus, standard preparticipation evaluation of athletes has a limited ability to detect these anomalies.

The Israeli experience showed neither utility nor cost effectiveness, despite the Israeli National Sports Law implementation in 1997. Steinvil et al [53] searched two newspapers to determine the yearly number of sudden cardiac death in Israeli athletes; he showed no difference in incidence of sudden cardiac death in athletes before or after electrocardiography screening. Reliance on newspapers can underestimate sudden cardiac death numbers; furthermore, assumptions regarding the number of screened athletes were only estimates. This lack of solid numbers for both the numerator and denominator makes the death rates not reliable. Moreover, it is a surprise that results of the Israel screening program were completely ignored with no information regarding the implementation of the national screening, the number of examined athletes, the proportion of disqualified ones, and the cardiac abnormalities discovered. In short, no data derived from the direct experience of sport physicians support the alleged inefficacy of the screening program in Israel.

None of the studies were primarily designed to prospectively evaluate electrocardiography in its ability to add value to history and physical examination, nor to determine the effect of the screening electrocardiography on overall cardiac outcome, survival, cost, and impact on athletes. The American Heart Association has been hesitant to adopt the international public health screening strategy for its young athletes [7] although electrocardiography screening is now mandatory in some professional organisations such as the National Basketball Association.

### 3.5 New 2010 electrocardiography criteria reduces false positive rate

The introduction of the 2010 European Society of Cardiology criteria for electrocardiography interpretation in athletes improved the false positive rate. When Corrado et al [47] applied this new classification to the previously reported data from Pelliccia et al [50] on 1005 highly trained athletes, 292 of the 402 athletes previously described as having electrocardiographic abnormalities showed either an isolated increase of QRS voltage (n = 233) or early repolarization patterns (n = 59). Only 11% (n = 110) were classified as “uncommon and training-unrelated” according to the new criteria. The new electrocardiographic criteria increased the electrocardiography specificity by 70%, primarily in the important group of athletes who exhibit pure voltage criteria for left ventricular hypertrophy and early repolarization abnormalities, maintaining sensitivity for detection of cardiovascular diseases at risk of sudden cardiac death during sports.

Similarly Weiner et al [54] examined the performance of the 2010 European Society of Cardiology criteria for electrocardiography interpretation in the same athletic population previously screened by Baggish et al using traditional criteria [45]. Application of the new electrocardiographic criteria improved the accuracy of an electrocardiographic-inclusive preparticipation screening strategy, by improving specificity [i.e. reducing the number of participants with false positive electrocardiography findings from 83 of 508 (16.3%) to 49 of 508 (9.6%) and, most importantly, preserving sensitivity]. The reduction in the number of abnormal electrocardiography was driven by the reclassification of participants with isolated QRS voltage criteria for left ventricular hypertrophy from abnormal to normal. This shows the potential efficacy of the new classification in increasing the specificity of electrocardiography as a part of cardiac screening.

It is imperative that only experienced sports cardiologist should perform electrocardiography, ultimately reducing the likelihood of recording false-positive or false-negative finding, thus reducing further investigation and hence improving the cost-effectiveness of this approach.

### 3.6 Is electrocardiography screening cost-effective?

Few data exist on the cost effectiveness of preparticipation cardiac screening in athletes, and these are not easily comparable. Wheeler et al [55] assessed the costs and survival rates in U.S. athletes who were screened with or without 12-lead ECG and estimated that electrocardiography resulted in 2.1 life-years saved per 1000 athletes screened. The cost-effectiveness ratio of the screening with electrocardiography was $42 000 per life-year saved. Fuller et al [56] showed similar results supportive of an electrocardiography based screening. It can be concluded that electrocardiography based screening is more cost-effective than history and physical examination alone ($8,800–$44,000 vs. >$84,000 respectively), with cost estimates per year of life saved below $50,000, which is the traditional threshold to consider a health intervention as cost-effective.

An Italian cost-effectiveness analysis of 33,375 athletes using a more conservative approach (10% of affected athletes would live an additional 20 years). The study estimated the cost per year of life saved at $18,666 for the Italian model.

The implementation of a national screening program to identify silent cardiac disease in healthy young athletes cannot be regarded cost-effective in the United Kingdom, were health service is already burdened with limited resources and finances. Perhaps further development of new even stricter electrocardiography criteria for abnormalities will have the potential to further decrease the false positive rate and potentially improving the cost-effectiveness.

### 3.7 Issue of sudden cardiac death incidence

Sudden cardiac death is the leading cause of death in young athletes. However, the exact incidence of sudden cardiac death is unknown, and it is difficult to compare studies with different methodology and from widely different geographic areas. A prospective Italian study showed an annual incidence of 1 in 25,000 young competitive athletes per year [34], while early American data of sudden cardiac death among high school and college athletes has been estimated to be <1 in 100,000 participant per year [1,57]. Each study differs in the methodology of data collection and athletic age group. In comparison to the American data, the Italian data included older athletes and a higher proportion of men (age range 12–35 vs. 12–24 years, men 85% vs. 65%). This can explain the higher reported incidence of mortality in the Italian study. Moreover, the Italian data were systematically collected from national registry; on the other hand, the American data were based on retrospective analysis of media report and insurance claims, which underestimate the mortality rate.

Studies with more accurate reporting system have found a higher incidence of sudden cardiac death than early estimates and quite similar to the Italian data. In National Collegiate Athletic Association athletes, the overall incidence of sudden cardiac death was 1:43,000 athletes per year. Higher risk was found in black male athletes (1:13,000) and male basketball players (1:7000) [2]. Similarly, a recent prospective population based study conducted at 11 American and Canadian cities found an incidence of sudden cardiac death from cardiovascular disease of 1:27 000 in young adults [58].

### 3.8 Limitations of the literature review

The access to only two search databases (MEDLINE and CINAHL) might have reduced the number of yielded searches and hence some articles might have not been detected, but a thorough search at references reduced this likelihood. The Downs and Black ‘Quality Index’ used was considered the most relevant quality assessment scale for evaluating case control and cohort studies. However many of the checklist were irrelevant, hence the power of the score was low. Results of this quality index should be taken cautiously as it was not conducted by independent reviewer.

## 4 Conclusion

The high false positive rate of electrocardiography, low prevalence of cardiac cause of sudden death, cost-effectiveness are all debated against with new data on high prevalence of sudden cardiac death in USA and improved false positive rate when using the new 2010 electrocardiography criteria from the European Society of Cardiology. The current scientific evidence suggests that screening with electrocardiography represents best clinical practice to prevent or reduce the risk of sudden cardiac death in young athletes. It significantly improves the sensitivity of the history and physical examination; it has reasonable specificity and excellent negative predictive value; and it is inexpensive compared to echocardiogram. Future studies must be conducted in a large, multicentre, multination, prospective trial that is powered to determine how different screening options affect the incidence of sudden cardiac death. While we await such studies one can recommend a targeted screening at certain age group and level of competition that will defiantly be a more cost-effective. Moreover, secondary prevention of sudden cardiac death is crucial with training the pitch side medical personnel on cardiac resuscitation and the immediate use of defibrillator.

## 



## Figures and Tables

**Table 1:** Search strategy and results from each included database

	**Key words**	**Medline**	**CINAHL**
**1**	Preparticipation	365	118
**2**	Screening	220644	27987
**3**	Athletes	18559	8797
**4**	Electrocardiography	94951	8372
**5**	Sudden, cardiac, death	5738	552
**6**	Preparticipation AND athletes (both exploded)	248	90
**7**	Preparticipation AND electrocardiography (both exploded)	70	23
**8**	Screening AND athletes (both exploded)	708	214
**9**	Screening AND electrocardiography (both exploded)	1620	231
**10**	Athletes AND electrocardiography (both exploded)	557	153
**11**	Athletes AND sudden cardiac death (both exploded)	227	28
**12**	6 AND electrocardiography	67	21
**13**	8 AND electrocardiography	158	38
**14**	11 AND electrocardiography	88	16
	Subtotal (12+13+14)	313	75
	Duplicate	114	19
	Total	199	56
	Total Search after combining all database search and removal of duplicate	226	

**Table 2:** Characteristics of studies evaluating the use of ECG in Athletes preparticipation evaluation

**Reference**	**Study Design**	**Study Setting/Measures**	**Conclusion**
Maron et al [29]	Cross sectional	501 Athletes from University of Maryland Hx, PE, and ECG compared to echo for evidence of CVD	Specificity 27%, false positive 15% Poor sensitivity, no cases of lethal CVD found. ECG did not increase sensitivity of Hx/PE
Corrado et al [30]	(1) Cohort (2) Cross sectional	Trend of SCD in athletes and nonathletic population (12–35yrs) in the Veneto region of Italy (period 1979–2004) Cardiovascular causes of sports disqualification in 42,386 athletes (period 1982–2004)	Decreased annual SCD by 89% 8.9% required further test (following ECG) 2% were disqualified
Fuller et al [31]	Cohort	5615 high school student athletes. Compared ECG to Hx, PE (by cardiologists and blinded) echo and stress test done as indicated	Specificity 97.8% for Hx/PE, 97.4% for ECG; ECG sensitivity 70%, false +ve rate 2.6% ECG has similar specificity to Hx/PE yet more effective as screening tool for CVD
Pelliccia et al [32]	Cross sectional	1005 elite Italian athletes from 38 sports. ECG patterns compared with echo (both interpreted blindly)	Sensitivity 51%, specificity 61%, PPV 7%, NPV 96% (for ECG detection) False positives caused by athletes heart limits ECG usefulness in PPE
Basso et al [33]	Retrospective case review	2 large registries of SCD in young athletes in USA and Italy. ECG, stress test, echo for detecting AOCA	27 cases of AOCA, age 9–32y, all had normal ECG, echo, stress test. Standard PPE limited in ability to detect
			AOCA
Baggish et al [34]	Cross sectional	510 collegiate athletes H/o, PE, with and without ECG	ECG improved sensitivity from 45.5% to 90,9%; NPV from 98.7% to 99.8%; False+ve 16.9%
Hevia et al [35]	Cross sectional	1220 Spanish athletes from different sports disciplines H/o, PE, ECG and further tests	3.7% required additional tests 2 diagnosed (1 echo, 1 MRI)
Magalski et al [36]	Cohort	964 competitive collegiate athletes H/o, PE, ECG and Echo	ECG improved sensitivity from 44.4% to 88.9%; NPV from 99.3% to 99.9%
Bessem et al [37]	Cross sectional	825 athletes cardiac screening using the Lausanne recommendation (H/o, PE, ECG) University centre of sports medicine in Groningen, Netherland	6.3% had additional test based on ECG ECG had 11% false positive rate Number needed to screen was 1:143
Sofi et al [38]	Cross sectional	30,065 participants in competitive sports at Institute of sports medicine in Florence, Italy H/o, PE, resting and stress ECG	Abnormal finding: Resting ECG 6% Stress ECG 4.9% 0.6% ineligible for competitive sports
Tanaka et al [39]	Prospective, cross sectional	37,804 students with 6 years follow up part of national cardiac screening program in Kagoshima, Japan (included athletes and non-athletes) H/x, PE, ECG, and echo ifneeded	3 SCD, one screened and diagnosed with HCM, 2 normal ECG findings Estimate cost of $8,800 per year of life saved
Marek et al [40]	Retrospective, cohort Study	High school ECG screening program (YH4L) in Chicago, USA, 32,561 High school student H/o, PE, ECG	2.5% had ECG abnormality requiring further test
Steinvil et al [41]	Retrospective, cohort study	Systematic search of 2 newspapers in Israel to determine number of SCD in competitive athletes. Israeli national mandatory PPE includes resting and stress ECG	2.6 events per 100,000 person-years ECG had no apparent influence on incidence of sudden death in athletes
Wilson et al [42]	Cross sectional	1074 nationaland international junior athletes and 1646 physical active schoolchildren H/o, PE and ECG (expert sports cardiologist)	4 WPW 3 Long QT 1 ARVC 1 Right ventricular outflow tract ventricular tachycardia Further tests in 4%
Pelliccia et al [43]	Cross sectional	4450 athletes of Italian national teams, eligible initially on ECG screening underwent echocardiography	No HCM Myocarditis(n=4) Mitral Valve Prolapse(n=3) Aortic regurgitation(n=2) ARVC(n=1)
Le et al [44]	Cross sectional	653 athletes from 24 sports at Stanford sports medicine program H/o, PE and ECG	10 % had abnormal ECG for further test

H/o: History, PE: Physical examination, ECG: electrocardiography, ARVC: arrythmogenic right ventricular cardiomyopathy, HCM: Hypertrophic cardiomyopathy, AOCA: Anomalous Origin of Coronary Artery, WPW: Wolf Parkinson White syndrome, SCD: Sudden cardiac death, CVD: cardiovascular disease, NPV: negative predictive value, PPV: Positive predictive value

**Table:** **Reporting**

**Study**	**Study design**	**Q1: Aim clearly described**	**Q2: Outcomes clearly described**	**Q3: Patients characteristics clearly described**	**Q4: Interventions clearly described**	**Q6: Main findings clearly described**	**Q7: Random variability for main outcome provided**	**Q9: Lost to follow up reported**	**Q10: Actual p-value reported**
**Maron et al [29]**	Cross sectional	Yes	Yes	Yes	Yes	Yes	Yes	No	No
**Corrado et al [30]**	Cohort	Yes	Yes	Yes	Yes	Yes	Yes	U	Yes
**Fuller et al [31]**	Cohort	Yes	Yes	Yes	Yes	Yes	Yes	Yes	No
**Pelliccia et al [32]**	Cross sectional	Yes	Yes	Yes	Yes	Yes	Yes	No	No
**Basso et al [33]**	Case review	Yes	Yes	Yes	Yes	Yes	Yes	Yes	Yes
**Baggish et al [34]**	Cross sectional	Yes	Yes	Yes	Yes	Yes	Yes	Yes	Yes
**Hevia et al [35]**	Cross sectional	Yes	Yes	Yes	Yes	Yes	Yes	Yes	No
**Magalski et al [36]**	Cohort	Yes	Yes	Yes	Yes	Yes	Yes	U	Yes
**Bessem et al [37]**	Cross sectional	Yes	Yes	Yes	Yes	Yes	Yes	Yes	Yes
**Sofi et al [38]**	Cross sectional	Yes	Yes	Yes	Yes	Yes	Yes	Yes	Yes
**Tanaka et al [39]**	Cross sectional	Yes	Yes	Yes	Yes	Yes	Yes	Yes	Yes
**Marek et al [40]**	Cohort study	Yes	Yes	Yes	Yes	Yes	Yes	No	Yes
**Steinvil et al [41]**	Cohort	Yes	Yes	Yes	Yes	Yes	No	No	Yes
**Wilson et al [42]**	Cross sectional	Yes	Yes	Yes	Yes	Yes	Yes	No	No
**Pelliccia et al [43]**	Cross sectional	Yes	Yes	Yes	Yes	Yes	Yes	Yes	No
**Le et al [44]**	Cross sectional	Yes	Yes	Yes	Yes	Yes	Yes	Yes	Yes

**Table:** External validity and Bias

**Study**	**Study design**	**Q11: Sample asked to participate representative of the population**	**Q12: Sample agreed to participate representative of the population**	**Q13: Staff participating representative of the patient’s environment**	**Q16: Data dredging results stated clearly**	**Q17: Analysis adjusted for length of follow up**	**Q18: Appropriate statistics**	**Q19: Reliable compliance**	**Q20: Accurate outcome measures**
Maron et al [29]	Cross sectional	Yes	Yes	U	Yes	No	Yes	Yes	Yes
Corrado et al [30]	Cohort	Yes	Yes	Yes	Yes	Yes	Yes	Yes	Yes
Fuller et al [31]	Cohort	Yes	Yes	Yes	Yes	U	U	Yes	U
Pelliccia et al [32]	Cross sectional	Yes	Yes	Yes	Yes	No	Yes	Yes	Yes
Basso et al [33]	Cross sectional	Yes	Yes	Yes	Yes	No	Yes	Yes	Yes
Baggish et al [34]	Cross sectional	Yes	Yes	Yes	Yes	No	Yes	Yes	Yes
Hevia et al [35]	Cross sectional	Yes	Yes	Yes	Yes	No	Yes	Yes	Yes
Magalski et al [36]	Cohort	Yes	Yes	Yes	Yes	U	Yes	Yes	Yes
Bessem et al [37]	Cross sectional	Yes	Yes	Yes	Yes	Yes	U	Yes	Yes
Sofi et al [38]	Cross sectional	Yes	Yes	Yes	Yes	Yes	Yes	Yes	Yes
Tanaka et al [39]	Cross sectional	U	Yes	U	No	U	Yes	U	Yes
Marek et al [40]	Cohort study	Yes	Yes	Yes	Yes	U	Yes	Yes	Yes
Steinvil et al [41]	Cohort study	U	U	U	U	U	No	No	No
Wilson et al [42]	Cross sectional	Yes	Yes	Yes	Yes	U	Yes	Yes	Yes
Pelliccia et al [43]	Cross sectional	Yes	Yes	Yes	No	U	Yes	Yes	Yes
Le et al [44]	Cross sectional	Yes	Yes	Yes	Yes	U	Yes	Yes	Yes

**Table:** Selection bias and power

**Study**	**Study design**	**Q21: Same population**	**Q22: Participants recruited at the same time**	**Q26: Loss of follow up reported?**	**Total score / 19**
Maron et al [29]	Cross sectional	Yes	Yes	No	14
Corrado et al [30]	Cohort	Yes	Yes	U	17
Fuller et al [31]	Cohort	Yes	Yes	Yes	15
Pelliccia et al [32]	Cross sectional	Yes	Yes	No	15
Basso et al [33]	Case review	Yes	Yes	No	17
Baggish et al [34]	Cross sectional	Yes	Yes	No	17
Hevia et al [35]	Cross sectional	Yes	No	No	15
Magalski et al [36]	Cohort	Yes	No	U	15
Bessem et al [37]	Cross sectional	Yes	No	No	16
Sofi et al [38]	Cross sectional	Yes	Yes	No	18
Tanaka et al [39]	Cross sectional	Yes	Yes	Yes	14
Marek et al [40]	Cohort study	Yes	Yes	U	16
Steinvil et al [41]	Cohort study	Yes	Yes	U	8
Wilson et al [42]	Cross sectional	Yes	Yes	No	15
Pelliccia et al [43]	Cross sectional	Yes	Yes	No	15
Le et al [44]	Cross sectional	Yes	Yes	Yes	18

Score of all questions Yes= 1, No = 0; U: Unable to determine

**Table 4:** Comparison of history and physical examination Vs ECG in screening young athletes

	**Positive results requiring further test**			**Sensitivity to Detect Lethal CVD**		
**Study**	**H&P**	**ECG**	**Total**	**No. of cases**	**H&P**	**ECG**
Wilson *et al* [42]	2.5%	1.5%	4%	9	0	100%
Bessem *et al* [37]	8%	8%	13%	3%	33%	67%
Hevia *et al* [35]	1.2%	6.1%	7.4%	2	0	100%
Baggish *et al* [34]	6%	16%	20%	3	33%	67%
**Total**	**4.4%**	**7.9%**	**11.1%**	**17**	**12%**	**88%**

H&P: History & Physical Examination
