# Supplementary material for: The Validity of Adding ECG to the Preparticipation Screening of Athletes An Evidence Based Literature Review
Source: Transl Med UniSa. 2014 Dec 19;11:2–13. (PMC4309649)
Supplement: Supplementary file 1 [file TM-11-02-s001.doc]

**Table 1:** Search strategy and results from each included database

|  | **Key words** | **Medline** | **CINAHL** |
| --- | --- | --- | --- |
| **1** | Preparticipation | 365 | 118 |
| **2** | Screening | 220644 | 27987 |
| **3** | Athletes | 18559 | 8797 |
| **4** | Electrocardiography | 94951 | 8372 |
| **5** | Sudden, cardiac, death | 5738 | 552 |
| **6** | Preparticipation AND athletes (both exploded) | 248 | 90 |
| **7** | Preparticipation AND electrocardiography (both exploded) | 70 | 23 |
| **8** | Screening AND athletes (both exploded) | 708 | 214 |
| **9** | Screening AND electrocardiography (both exploded) | 1620 | 231 |
| **10** | Athletes AND electrocardiography (both exploded) | 557 | 153 |
| **11** | Athletes AND sudden cardiac death (both exploded) | 227 | 28 |
| **12** | 6 AND electrocardiography | 67 | 21 |
| **13** | 8 AND electrocardiography | 158 | 38 |
| **14** | 11 AND electrocardiography | 88 | 16 |
|  | Subtotal ( 12+13+14 ) | 313 | 75 |
|  | Duplicate | 114 | 19 |
|  | Total | 199 | 56 |
|  | Total Search after combining all database search and removal of duplicate | 226 | |

**Table 2: Characteristics of studies evaluating the use of ECG in Athletes preparticipation evaluation**

| **Reference** | **Study Design** | **Study Setting/Measures** | **Conclusion** |
| --- | --- | --- | --- |
| Maron et al(29) | Cross sectional | 501 Athletes from University of Maryland  Hx, PE, and ECG compared to echo for evidence of CVD | Specificity 27%, false positive 15%  Poor sensitivity, no cases of lethal CVD found. ECG did not increase sensitivity of Hx/PE |
| Corrado et al(30) | (1) Cohort  (2) Cross sectional | Trend of SCD in athletes and nonathletic population (12-35yrs) in the Veneto region of Italy (period 1979-2004)  Cardiovascular causes of sports disqualification in 42,386 athletes (period 1982-2004) | Decreased annual SCD by 89%  8.9% required further test (following ECG)  2% were disqualified |
| Fuller et al(31) | Cohort | 5615 high school student athletes.  Compared ECG to Hx, PE (by cardiologists and blinded) echo and stress test done as indicated | Specificity 97.8% for Hx/PE, 97.4% for ECG; ECG sensitivity 70%, false +ve rate 2.6%  ECG has similar specificity to Hx/PE yet more effective as screening tool for CVD |
| Pelliccia et al(32) | Cross sectional | 1005 elite  Italian athletes from 38 sports.  ECG patterns compared with echo ( both interpreted blindly) | Sensitivity 51%, specificity 61%, PPV 7%, NPV 96% ( for ECG detection)  False positives caused by athletes heart limits ECG usefulness in PPE |
| Basso et al(33) | Retrospective case review | 2 large registries of SCD in young athletes in USA and Italy.  ECG, stress test, echo for detecting AOCA | 27 cases of AOCA, age 9-32y, all had normal ECG, echo, stress test.  Standard PPE limited in ability to detect AOCA |
| Baggish et al(34) | Cross sectional | 510 collegiate athletes  H/o, PE, with and without ECG | ECG improved sensitivity from 45.5% to 90,9%; NPV from 98.7% to 99.8%; False +ve 16.9% |

**Table 2. Continued**

| **Reference** | **Study Design** | **Study Setting/ Measures** | **Conclusion** |
| --- | --- | --- | --- |
| Hevia et al(35) | Cross sectional | 1220 Spanish athletes from different sports disciplines  H/o, PE, ECG and further tests | 3.7% required additional tests  2 diagnosed (1 echo, 1 MRI) |
| Magalski et al(36) | Cohort | 964 competitive collegiate athletes  H/o, PE, ECG and Echo | ECG improved sensitivity from 44.4% to 88.9%; NPV from 99.3% to 99.9% |
| Bessem et al(37) | Cross sectional | 825 athletes cardiac screening using the Lausanne recommendation (H/o, PE, ECG)  University centre of sports medicine in Groningen, Netherland | 6.3% had additional test based on ECG  ECG had 11% false positive rate  Number needed to screen was 1:143 |
| Sofi et al(38) | Cross sectional | 30,065 participants in competitive sports at Institute of sports medicine in Florence , Italy  H/o, PE, resting and stress ECG | Abnormal finding:  Resting ECG 6%  Stress ECG 4.9%  0.6% ineligible for competitive sports |
| Tanaka et al(39) | Prospective,  cross sectional | 37,804 students with 6 years follow up part of national cardiac screening program in Kagoshima, Japan  ( included athletes and non-athletes)  H/x, PE, ECG, and echo if needed | 3 SCD, one screened and diagnosed with HCM, 2 normal ECG findings  Estimate cost of $8,800 per year of life saved |
| Marek et al(40) | Retrospective,  cohort Study | High school ECG screening program (YH4L) in Chicago, USA, 32,561 High school student  H/o, PE, ECG | 2.5% had ECG abnormality requiring further test |

**Table 2. Continued**

| **Reference** | **Study Design** | **Study Setting/ Measures** | **Conclusion** |
| --- | --- | --- | --- |
| Steinvil et al(41) | Retrospective, cohort study | Systematic search of 2 newspapers in Israel to determine number of SCD in competitive athletes.  Israeli national mandatory PPE includes resting and stress ECG | 2.6 events per 100,000 person-years  ECG had no apparent influence on incidence of sudden death in athletes |
| Wilson et al(42) | Cross sectional | 1074 national and international junior athletes and 1646 physical active schoolchildren  H/o, PE and ECG ( expert sports cardiologist) | 4 WPW  3 Long QT  1 ARVC  1 Right ventricular outflow tract ventricular tachycardia  Further tests in 4% |
| Pelliccia et al(43) | Cross sectional | 4450 athletes of Italian national teams, eligible  initially on ECG screening underwent echocardiography | No HCM  Myocarditis(n=4)  Mitral Valve Prolapse(n=3)  Aortic regurgitation(n=2)  ARVC(n=1) |
| Le et al(44) | Cross sectional | 653 athletes from 24 sports at Stanford sports medicine program  H/o, PE and ECG | 10 % had abnormal ECG for further test |

*H/o: History, PE: Physical examination, ECG: electrocardiography, ARVC: arrythmogenic right ventricular cardiomyopathy, HCM: Hypertrophic cardiomyopathy, AOCA: Anomalous Origin of Coronary Artery, WPW: Wolf Parkinson White syndrome, SCD: Sudden cardiac death, CVD: cardiovascular disease, NPV: negative predictive value, PPV: Positive predictive value*

Table 3: Modified Downs & Black(28) quality index results of all included studies

## Reporting

| **Study** | **Study design** | **Q1: Aim clearly described** | **Q2: Outcomes clearly described** | **Q3: Patients characteristics clearly described** | **Q4: Interventions clearly described** | **Q6: Main findings clearly described** | **Q7: Random variability for main outcome provided** | **Q9: Lost to follow up reported** | **Q10: Actual p-value reported** |
| --- | --- | --- | --- | --- | --- | --- | --- | --- | --- |
| **Maron et al(29)** | Cross sectional | Yes | Yes | Yes | Yes | Yes | Yes | No | No |
| **Corrado et al(30)** | Cohort | Yes | Yes | Yes | Yes | Yes | Yes | U | Yes |
| **Fuller et al(31)** | Cohort | Yes | Yes | Yes | Yes | Yes | Yes | Yes | No |
| **Pelliccia et al(32)** | Cross sectional | Yes | Yes | Yes | Yes | Yes | Yes | No | No |
| **Basso et al(33)** | Case review | Yes | Yes | Yes | Yes | Yes | Yes | Yes | Yes |
| **Baggish et al(34)** | Cross sectional | Yes | Yes | Yes | Yes | Yes | Yes | Yes | Yes |
| **Hevia et al(35)** | Cross sectional | Yes | Yes | Yes | Yes | Yes | Yes | Yes | No |
| **Magalski et al(36)** | Cohort | Yes | Yes | Yes | Yes | Yes | Yes | U | Yes |
| **Bessem et al(37)** | Cross sectional | Yes | Yes | Yes | Yes | Yes | Yes | Yes | Yes |
| **Sofi et al(38)** | Cross sectional | Yes | Yes | Yes | Yes | Yes | Yes | Yes | Yes |
| **Tanaka et al(39)** | Cross sectional | Yes | Yes | Yes | Yes | Yes | Yes | Yes | Yes |
| **Marek et al(40)** | Cohort study | Yes | Yes | Yes | Yes | Yes | Yes | No | Yes |
| **Steinvil et al(41)** | Cohort | Yes | Yes | Yes | Yes | Yes | No | No | Yes |
| **Wilson et al(42)** | Cross sectional | Yes | Yes | Yes | Yes | Yes | Yes | No | No |
| **Pelliccia et al(43)** | Cross sectional | Yes | Yes | Yes | Yes | Yes | Yes | Yes | No |
| **Le et al (44)** | Cross sectional | Yes | Yes | Yes | Yes | Yes | Yes | Yes | Yes |

## Table 3: .Continued

## External validity and Bias

| **Study** | **Study design** | **Q11: Sample asked to participate representative of the population** | **Q12: Sample agreed to participate representative of the population** | **Q13: Staff participating representative of the patient's environment** | **Q16: Data dredging results stated clearly** | **Q17: Analysis adjusted for length of follow up** | **Q18: Appropriate statistics** | **Q19: Reliable compliance** | **Q20: Accurate outcome measures** |
| --- | --- | --- | --- | --- | --- | --- | --- | --- | --- |
| **Maron et al(29)** | Cross sectional | Yes | Yes | U | Yes | No | Yes | Yes | Yes |
| **Corrado et al(30)** | Cohort | Yes | Yes | Yes | Yes | Yes | Yes | Yes | Yes |
| **Fuller et al(31)** | Cohort | Yes | Yes | Yes | Yes | U | U | Yes | U |
| **Pelliccia et al(32)** | Cross sectional | Yes | Yes | Yes | Yes | No | Yes | Yes | Yes |
| **Basso et al(33)** | Cross sectional | Yes | Yes | Yes | Yes | No | Yes | Yes | Yes |
| **Baggish et al(34)** | Cross sectional | Yes | Yes | Yes | Yes | No | Yes | Yes | Yes |
| **Hevia et al(35)** | Cross sectional | Yes | Yes | Yes | Yes | No | Yes | Yes | Yes |
| **Magalski et al(36)** | Cohort | Yes | Yes | Yes | Yes | U | Yes | Yes | Yes |
| **Bessem et al(37)** | Cross sectional | Yes | Yes | Yes | Yes | Yes | U | Yes | Yes |
| **Sofi et al(38)** | Cross sectional | Yes | Yes | Yes | Yes | Yes | Yes | Yes | Yes |
| **Tanaka et al(39)** | Cross sectional | U | Yes | U | No | U | Yes | U | Yes |
| **Marek et al(40)** | Cohort study | Yes | Yes | Yes | Yes | U | Yes | Yes | Yes |
| **Steinvil et al(41)** | Cohort study | U | U | U | U | U | No | No | No |
| **Wilson et al(42)** | Cross sectional | Yes | Yes | Yes | Yes | U | Yes | Yes | Yes |
| **Pelliccia et al(43)** | Cross sectional | Yes | Yes | Yes | No | U | Yes | Yes | Yes |
| **Le et al(44)** | Cross sectional | Yes | Yes | Yes | Yes | U | Yes | Yes | Yes |

## Table 3: .Continued

## *Selection bias and power*

| **Study** | **Study design** | **Q21: Same population** | **Q22: Participants recruited at the same time** | **Q26: Loss of follow up reported?** | **Total score / 19** |
| --- | --- | --- | --- | --- | --- |
| **Maron et al(29)** | Cross sectional | Yes | Yes | No | 14 |
| **Corrado et al(30)** | Cohort | Yes | Yes | U | 17 |
| **Fuller et al(31)** | Cohort | Yes | Yes | Yes | 15 |
| **Pelliccia et al(32)** | Cross sectional | Yes | Yes | No | 15 |
| **Basso et al(33)** | Case review | Yes | Yes | No | 17 |
| **Baggish et al(34)** | Cross sectional | Yes | Yes | No | 17 |
| **Hevia et al(35)** | Cross sectional | Yes | No | No | 15 |
| **Magalski et al(36)** | Cohort | Yes | No | U | 15 |
| **Bessem et al(37)** | Cross sectional | Yes | No | No | 16 |
| **Sofi et al(38)** | Cross sectional | Yes | Yes | No | 18 |
| **Tanaka et al(39)** | Cross sectional | Yes | Yes | Yes | 14 |
| **Marek et al(40)** | Cohort study | Yes | Yes | U | 16 |
| **Steinvil et al(41)** | Cohort study | Yes | Yes | U | 8 |
| **Wilson et al(42)** | Cross sectional | Yes | Yes | No | 15 |
| **Pelliccia et al(43)** | Cross sectional | Yes | Yes | No | 15 |
| **Le et al(44)** | Cross sectional | Yes | Yes | Yes | 18 |

*Score of all questions (except Q 5) Yes= 1, No = 0; for question 5: Yes=2, Partial=1, No=0, U: Unable to determine*

**Table 4:** Comparison of history and physical examination Vs ECG in screening young athletes

|  | **Positive results requiring further test** | | | **Sensitivity to Detect Lethal CVD** | | |
| --- | --- | --- | --- | --- | --- | --- |
| **Study** | **H&P** | **ECG** | **Total** | **No. of cases** | **H&P** | **ECG** |
| **Wilson *et al*(42)** | 2.5% | 1.5% | 4% | 9 | 0 | 100% |
| **Bessem *et a*l(37)** | 8% | 8% | 13% | 3% | 33% | 67% |
| **Hevia *et al*(35)** | 1.2% | 6.1% | 7.4% | 2 | 0 | 100% |
| **Baggish *et al*(34)** | 6% | 16% | 20% | 3 | 33% | 67% |
| **Total** | **4.4%** | **7.9%** | **11.1%** | **17** | **12%** | **88%** |
